# The first observation of 4D tomography measurement of plasma structures and fluctuations

**DOI:** 10.1038/s41598-021-83191-3

**Published:** 2021-02-19

**Authors:** Chanho Moon, Kotaro Yamasaki, Yoshihiko Nagashima, Shigeru Inagaki, Takeshi Ido, Takuma Yamada, Naohiro Kasuya, Yusuke Kosuga, Makoto Sasaki, Yuichi Kawachi, Daiki Nishimura, Taiki Kobayashi, Akihide Fujisawa

**Affiliations:** 1 Research Institute for Applied Mechanics, Kyushu University, 6-1 Kasuga-Koen, Kasuga-City, Fukuoka 816-8580 Japan; 2 Faculty of Arts and Science, Kyushu University, 744 Motooka, Nishi-ku, Fukuoka 819-0395 Japan; 3 Interdisciplinary Graduate School of Engineering Sciences, Kyushu University, 6-1 Kasuga-Koen, Kasuga-City, 816-8580 Japan; 4 Present Address: Department of Mechanical Engineering, Graduate School of Engineering, Hiroshima University, Higashihiroshima, Hiroshima 739-8527 Japan

**Keywords:** Plasma physics, Magnetically confined plasmas

## Abstract

A tomography system is installed as one of the diagnostics of new age to examine the three-dimensional characteristics of structure and dynamics including fluctuations of a linear magnetized helicon plasma. The system is composed of three sets of tomography components located at different axial positions. Each tomography component can measure the two-dimensional emission profile over the entire cross-section of plasma at different axial positions in a sufficient temporal scale to detect the fluctuations. The four-dimensional measurement including time and space successfully obtains the following three results that have never been found without three-dimensional measurement: (1) in the production phase, the plasma front propagates from the antenna toward the end plate with an ion acoustic velocity. (2) In the steady state, the plasma emission profile is inhomogeneous, and decreases along the axial direction in the presence of the azimuthal asymmetry. Furthermore, (3) in the steady state, the fluctuations should originate from a particular axial position located downward from the helicon antenna.

## Introduction

It is essential to understand the mechanism of fluctuation-driven transport in magnetically confined plasmas^1,2^. The transport is mainly dominated by plasma turbulence which consists of microscale^3^, mesoscale^4^, and macroscale fluctuations^5^, and the plasma turbulence has intrinsically nonlinear interactions between the disparate scale fluctuations. Furthermore, it has been pointed out that a turbulence asymmetry or a localization is on the magnetic field flux surface, which is reported in Refs.^6–8^, should considerably affect plasma transport. However, a direct and simultaneous observation of the entire structure with the disparate scale fluctuations is difficult by the conventional diagnostics, which only provide a measurement of local turbulence in a limited region of plasma cross-section^9–11^.

To examine the phenomena related to symmetry breaking, a diagnostic is indispensable to simultaneously observe the two-dimensional (2-D) structure and dynamics of the turbulence. Computed tomography is a promising approach for measuring the entire structure and the disparate scale of plasma fluctuations^12^. Tomography has been already used as plasma diagnostics to cylindrical and toroidal devices^13–25^, although the past and existing tomography applications have never detected the plasma fluctuations in sufficient temporal scales. Specifically, 2-D tomography has been constructed in the plasma assembly for nonlinear turbulence analysis (PANTA) device^26^. At present the 2-D tomography system has succeeded in measuring the entire plasma cross-section perpendicular to the magnetic field line with the development of many useful analysis techniques for obtained tomography images^27–30^.

Specifically, it has been found that the inhomogeneity along the magnetic field should play a role in structural formation in magnetized plasma^31^. Hence, the 2-D tomography is insufficient to obtain a more comprehensive understanding of the entire field of turbulence. Therefore, a new three-dimensional (3-D) tomography is developed to more specifically examine the complexity of plasma turbulence structure both in parallel and perpendicular direction to magnetic field, the 3-D tomography system is expected to help study the mechanism of plasma production during which the plasma could be intrinsically inhomogeneous, particularly using a helicon source^32–34^. This study presents the tomography system that achieve, for the first time, 3-D measurement with a sufficient temporal resolution to be able to observe the fluctuation dynamics, and the new experimental results and discoveries of a linear magnetized plasma in PANTA.

## Experimental apparatus and 3-D tomography systems

**Figure 1:**
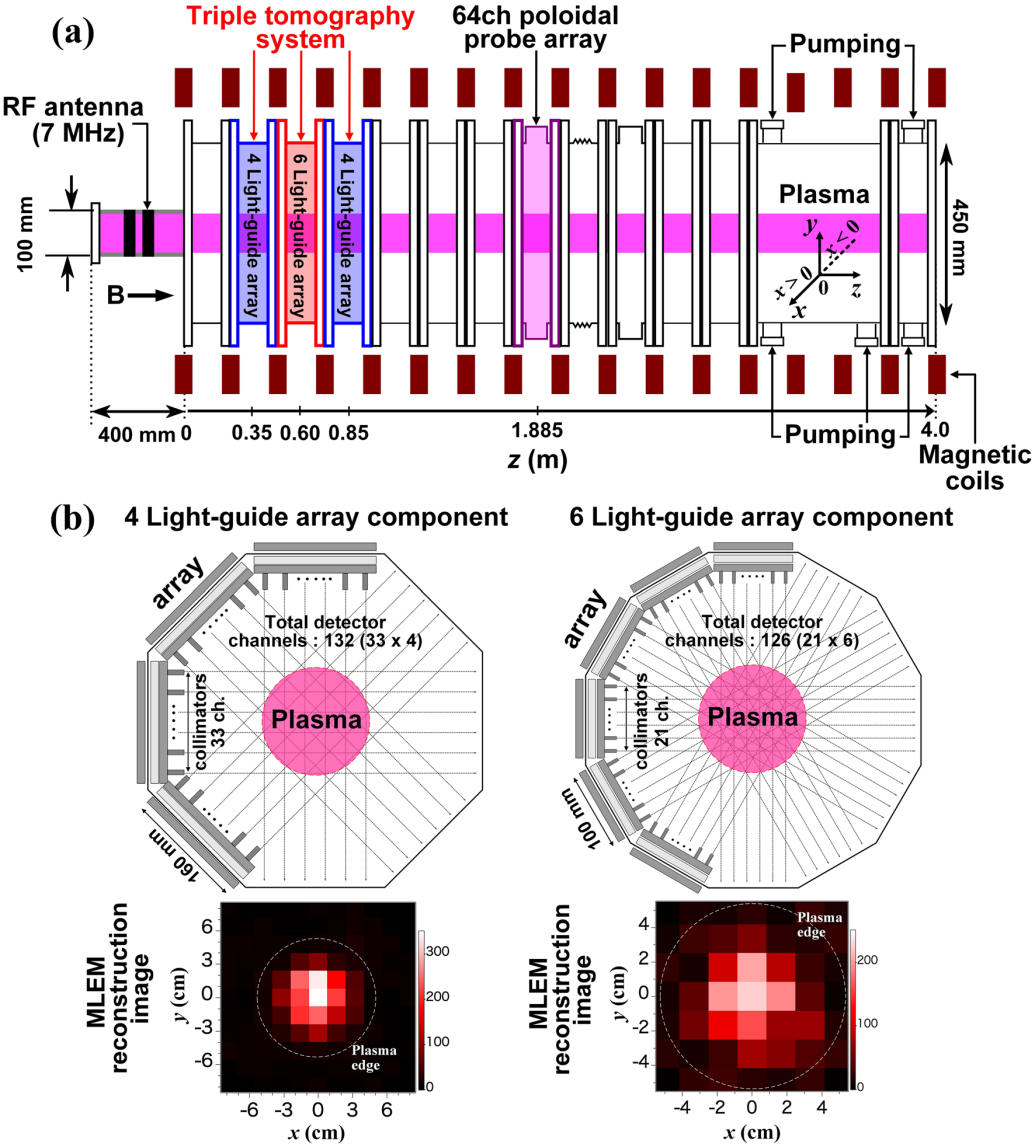
(**a**) Schematic diagram of the cylindrical magnetized plasma device PANTA. (**b**) The tomography system is composed of three components with four sets and six sets of light-guide array. The plasma images are reconstructed using MLEM.

The 3-D tomography system was developed in a linear magnetized apparatus, PANTA^26^, which is schematically shown in Fig. 1a. The vacuum vessel has a cylindrical shape whose length and diameter are 4.0 m and 0.45 m, respectively. In PANTA, a double loop antenna winded around the quartz tube (100 mm diameter and 400 mm axial length) is used to produce linear argon plasmas by the helicon wave whose maximum power and frequency are up to 6.0 kW and 7 MHz, respectively. As shown in the figure, here *x*-, *y*-, and *z*-coordinates indicate the horizontal, the vertical, and the axial directions, respectively.

In the PANTA device, the first trial of the tomography for turbulence measurement was made and succeeded in measuring the entire cross-section of plasma structure and fluctuations^12,27–31^. Specifically, two other similar tomography components have been recently installed on PANTA; the new tomography system, which is composed of three components, can perform 3-D imaging capabilities to more specifically examine the structure of plasma and its turbulence. The schematic diagram of the 3-D tomography system with four and six sets of light-guide arrays and the reconstruction plasma images using the tomography algorithm named maximum likelihood–expectation maximization (MLEM)^35^ are shown in Fig. 1b. Here, the MLEM method is chosen since it has no a priori assumption in the reconstruction algorithm^12^. The two components of the tomography system are equipped with four sets of light-guide arrays^12^ located at located at *z* = 0.35 m and *z* = 0.85 m; each one has 132 channels to detect line-integrated emission signals of plasma from =  − 80 to 80 mm, where *L* means the distance of line of sight from the device center (corresponding to plasma center). The light-guide arrays, with 33 channels each, are installed on different azimuthal angle positions that are separated by 45°. Furthermore, the other tomography component with six sets of light-guide array is located at *z* = 0.60 m, which has 126 channels to observe the region from *L* =  − 50 to 50 mm. The light-guide arrays, with 21 channels each, are installed on different azimuthal angle positions separated by 30°. Therefore, the later component provides higher spatial resolution of plasma emission image than the former components with four sets of light-guide array^36^.

## Experimental results and discussion

In this experiment, 3 kW RF power and 0.5 Pa argon gas pressure (*P*_Ar_) are set as the operation conditions, and the helicon plasma is confined by a homogeneous axial magnetic field of *B* = 0.09 T. The operation ranges of *P*_Ar_ and *B* in PANTA are 0.01–0.1 Pa and 0.01–0.13 T, respectively. The pulsed plasma discharge duration time is 600 ms in the experiment.

**Figure 2:**
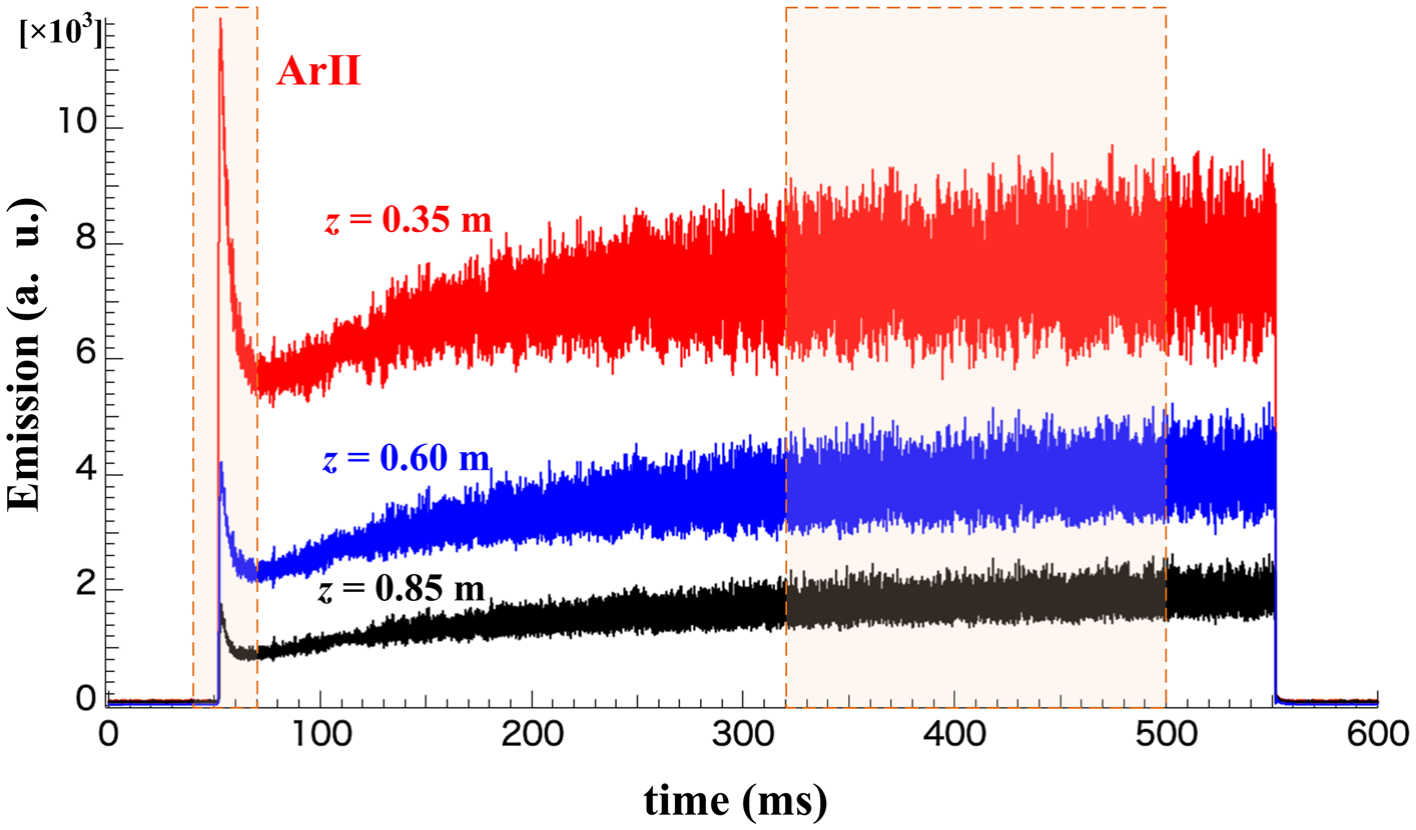
Overview of temporal evolutions of the total ArII emission (filling pressure = 0.5 Pa) at the axial positions of *z* = 0.35, 0.60, and 0.85 m. The highlight marks indicate the production and relaxation phase, and the quasi-steady state in the discharge.

The temporal evolution of total ArII emissions, ${\epsilon }_{total}(z)$, at the three axial positions, *z* = 0.35, 0.60, and 0.85 m, as an overview of the discharge is shown in Fig. 2. The temporal evolutions of the entire discharge show that plasma emission starts with a rapid increase in the plasma production phase at every axial position, which is followed by the relaxation phase to the steady state represented by constant emission. Furthermore, the substantial difference in emissivity is observed during the discharge; the emission clearly decreased with an increase in the axial distance from the antenna. The observation implies the inhomogeneity in the density along the magnetic field.

**Figure 3:**
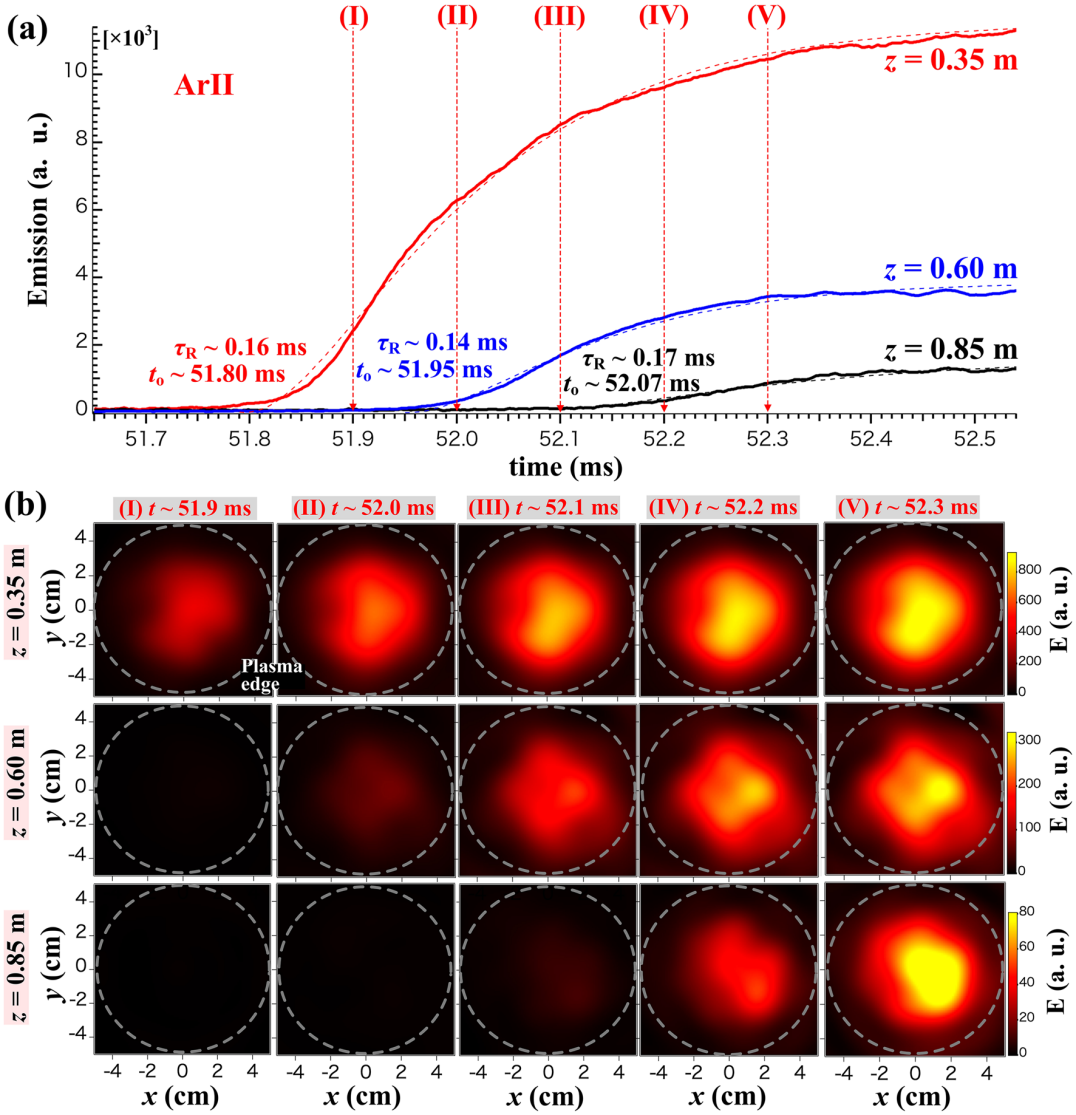
(**a**) Temporal evolution of the total emission and (**b**) two-dimensional images of emission at the three axial positions during the plasma production phase, *z* = 0.35, 0.60, and 0.85 m. The images are obtained using the FBF fitted to MLEM profile. The dashed circles indicate the plasma boundary.

**Figure 4:**
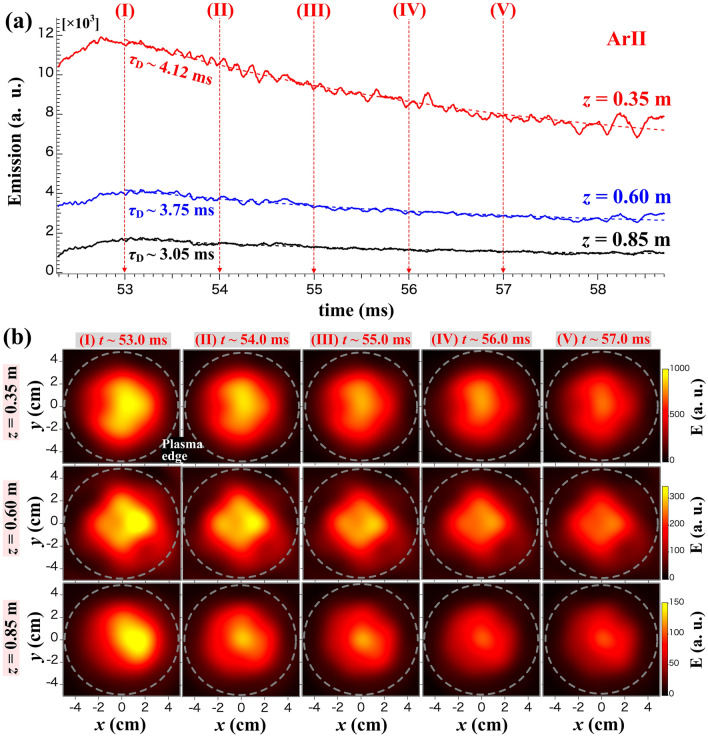
(**a**) Temporal evolution of the total emission and (**b**) two-dimensional images of emission at the three axial positions during the relaxation phase, *z* = 0.35, 0.60, and 0.85 m. The images are obtained using the FBF fitted to MLEM profile. The dashed circles indicate the plasma boundary.

First, the behavior of emission intensity is examined during the plasma production phase (*t* ≃ 51.7–52.5 ms). An expanded view of the temporal evolution of total emission (${\epsilon }_{total}$) at the three axial positions is shown in Fig. 3a. The characteristic time constants of the evolution are evaluated by fitting an exponential function from, $\in_{total} \propto \left[ {1 - \exp \left( { - \left( {\frac{{t - t_{0} }}{{\tau_{R} }}} \right)} \right)} \right]^{2}$, where *t*_0_ and ${\tau }_{R}$ are the start rising time and the rising time scale. The results are obtained as ${(t}_{0},{\tau }_{R})\simeq$ (51.80 ms, 0.16 ms), (51.95 ms, 0.14 ms), and (52.07 ms, 0.17 ms), for *z* = 0.35, 0.60, and 0.85 m, respectively. It is determined that the *t*_0_ is delayed because the axial position is more distant from the antenna, although the ${\tau }_{R}$ is in the range of a few hundred microseconds for every axial position. The observation shows the instantaneous emissions because the plasma front should move downward just after the plasma production in the antenna. The propagation velocity in the z-axis is approximately consistent with the ion acoustic velocity (~ 1.85 km/s). The velocity is calculated from the propagated distance and the difference in the *t*_0_. Furthermore, the reconstructed images are obtained using the Fourier–Bessel functions (FBF) fitted to the emission profile image after the reconstruction using the MLEM method^27^, where the dashed circles indicate the plasma boundary corresponding to the circular shape of the antenna to produce the plasma, as is shown in Fig. 3b. The FBF images show a delay in the rising time with detailed difference in emission profiles. These features are especially expected to be great help to understand the physics of plasm production. On the other hand, the temporal evolution of ${\epsilon }_{total}$ at the three axial positions during the relaxation phase (*t* ≃ 52–59 ms) is shown in an expanded view of Fig. 4a. The decay time, ${\tau }_{D}$, are evaluated, by fitting an exponential function from, ${\epsilon }_{total}\left(t\right)={\epsilon }_{total,0}\mathrm{exp}(-t/{\tau }_{D})$, as ${\tau }_{D}\simeq 4.12$, 3.75, and 3.05 ms, for *z* = 0.35, 0.60, and 0.85 m, respectively. The FBF images in Fig. 4b shows the detailed changes of entire plasma emission.

**Figure 5:**
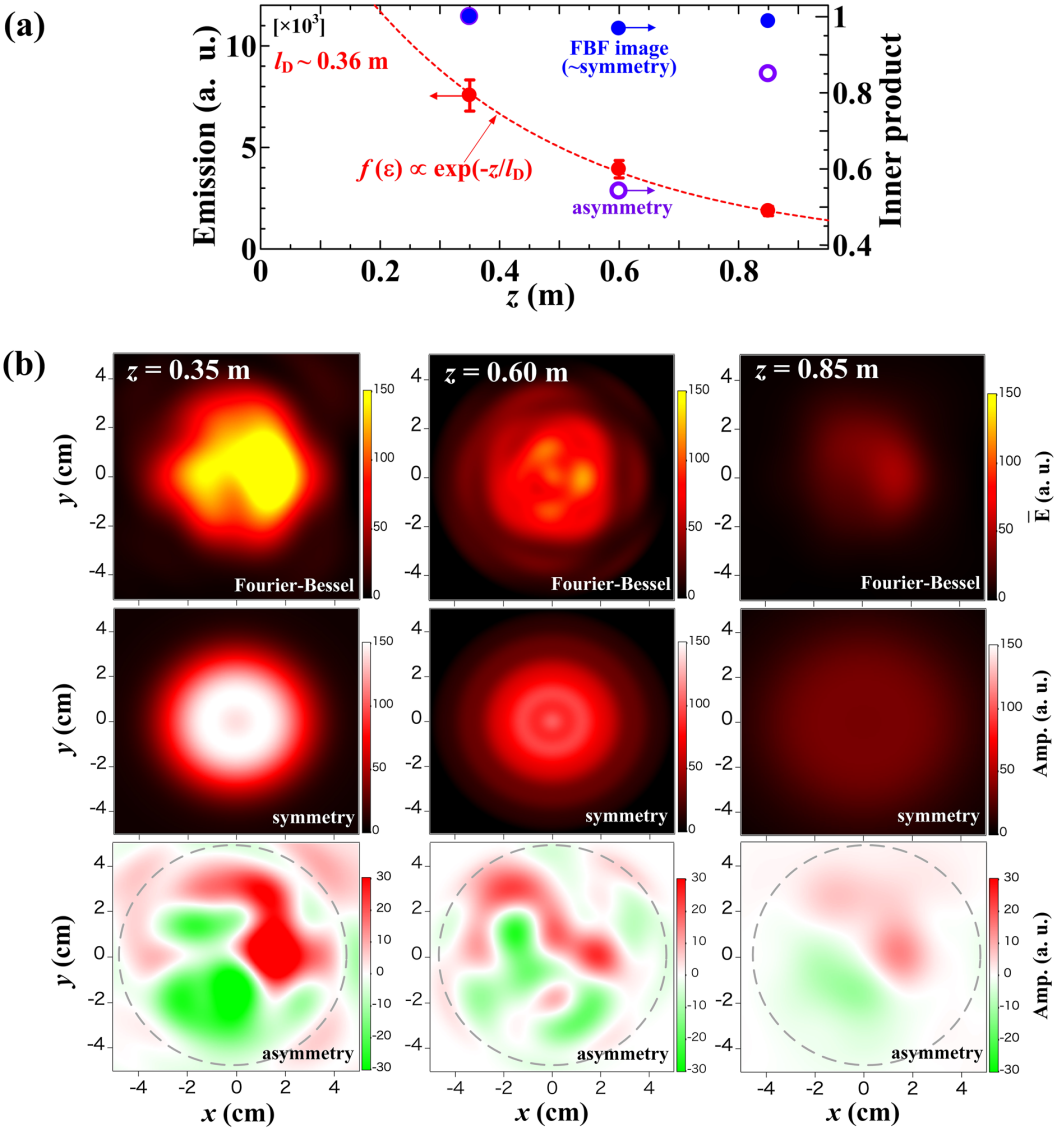
(**a**) Dependence of the total emission and the inner product on the axial position and (**b**) the FBF images at the axial position of *z* = 0.35, 0.60, and 0.85 m. The fitting images are divided into the symmetrical and asymmetrical part. The results are calculated in the quasi-steady state (*t* = 0.32–0.50 s).

The 3-D characteristics of emission are examined for the steady state (*t* = 0.32–0.50 s, ∆*t* ~ 0.18 s), where the emission can be regarded as being a constant. The axial dependence of the total emission intensity and the similarity of the structure evaluated by the inner product coefficient is shown in Fig. 5a. It is determined that the total emission considerably decreased along the *z*-axis because of the recombination of argon ions with the neutrals under the operational condition of rather high filling gas pressure (*P*_Ar_ = 0.5 Pa). For this experimental condition, the decay length, ${l}_{D}$, is evaluated as ${l}_{D}\simeq 0.36$ m, where the decay length is determined to obey the expression, ${\epsilon }_{total}\left(z\right)={\epsilon }_{total,0}\mathrm{exp}(-z/{l}_{D})$. It is determined that the ${l}_{D}$ is much shorter than the device length of 4 m. For PANTA plasmas, it is known that the electron temperature should show no significant change and remain constant at approximately 3 eV everywhere^37^. Furthermore, the emission intensity is approximately proportional to *n*_e_^2^^17,38^; therefore, the ${l}_{D}$ of density is expected to be twice as large as the above-mentioned value, i.e., approximately 0.7 m.

The similarity of the structure is quantitatively evaluated with the inner product of the local FBF image data, which is defined in the following expression

$$\xi \left({z}_{1}, {z}_{i}\right)=\frac{\sum_{x,y}\epsilon \left(x,y,{z}_{1}\right)\epsilon \left(x,y,{z}_{i}\right)}{\left|\epsilon \left(x,y,{z}_{1}\right)\right|\left|\epsilon \left(x,y,{z}_{i}\right)\right|}.$$

where *i* (1–3) and $\epsilon \left(x,y,{z}_{1}\right)$ present the order of the axial positions and the FBF image data at *z*_1_ = 0.35 m, respectively. Supposed that the set of the emission values $\epsilon \left(x,y,{z}_{1}\right)$ should be regarded as a vector, the numerator of the above equation corresponds to the inner product of the vectors of the two local emission values. The inner product $\xi \left({z}_{1}, {z}_{i}\right)$ provides the means of defining orthogonality between the 2D structural images. The evaluation shows that the whole FBF image structure should be almost the same in the axial range of the observation, but the asymmetrical part corresponding to plasma fluctuation tends to drastically decrease at *z* = 0.60 m. The details are shown in Fig. 5b, which plots the 2-D emission profiles at three different positions, i.e., *z* = 0.35, 0.60, and 0.85 m. The profiles are obtained using the FBF fitted to the temporal averaged MLEM profile (*t* = 0.32–0.50 s, ∆*t* ~ 0.18 s)^27^. It is determined that asymmetry should exist for the emission profiles at three axial positions, although the plasma device is assumed to be symmetric. The symmetrical and asymmetrical parts indicate the FBF images composed of only azimuthal mode number *m* = 0 and the other modes ($m\ne 0$). The asymmetrical part of the emission profile definitely changes along the *z*-axis, although the tomographic image can be finer at *z* = 0.60 m because of the high spatial resolution of six sets of light-guide array^36^.

**Figure 6:**
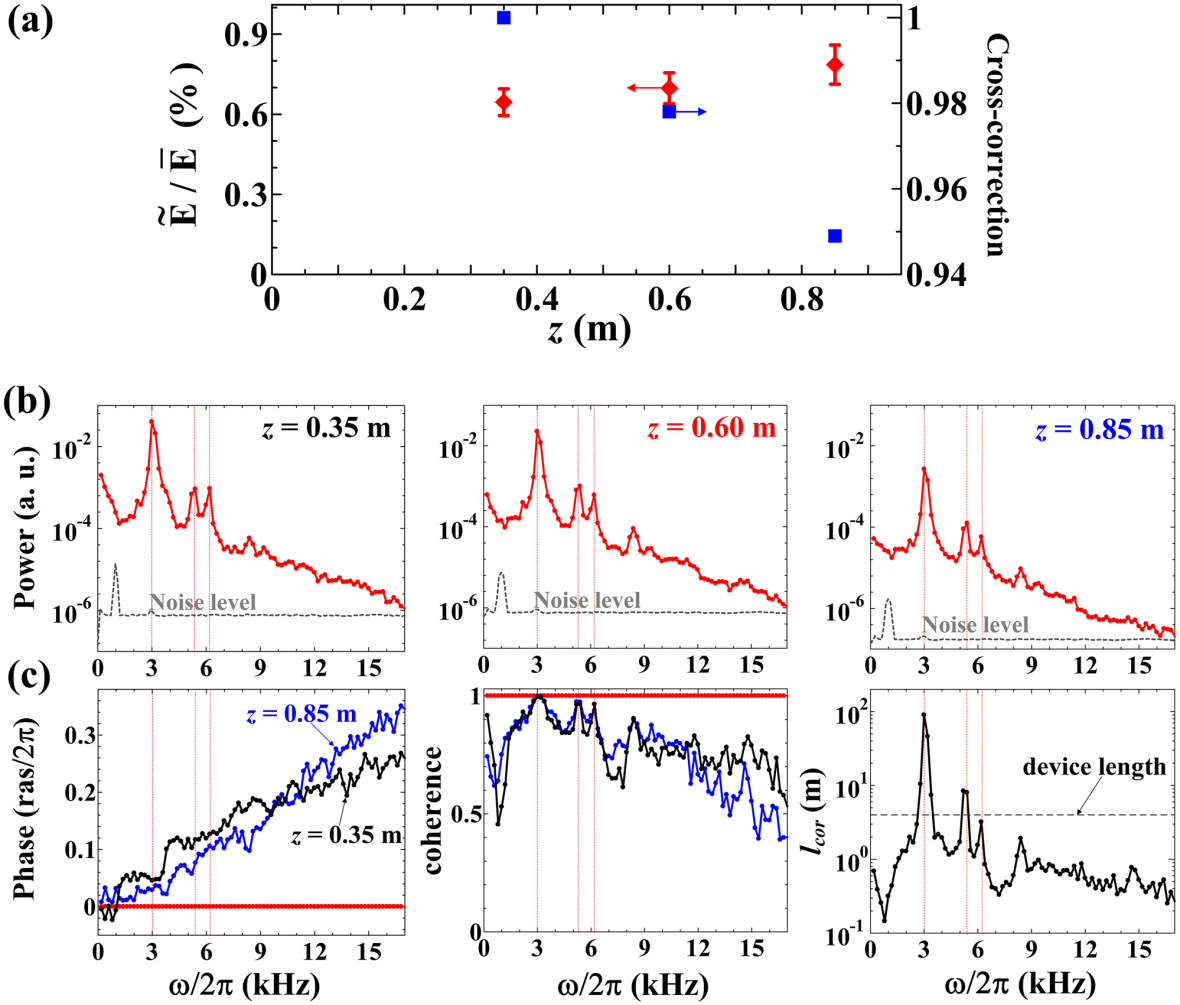
(**a**) Axial dependence of the normalized amplitude of total emission fluctuations (*f* ≤ 20 kHz) and the cross-correction. (**b**) Fluctuation spectra of the total plasma emission at the axial positions of *z* = 0.35, 0.60, and 0.85 m. (**c**) Axial cross-phase, coherence, and the correlation length (*l*_cor_) with reference position at *z* = 0.60 m. The calculations are performed in the quasi-steady state (*t* = 0.32–0.50 s).

The axial dependence of normalized fluctuation amplitude and cross-correction, which is calculated for ∆*t* ~ 0.18 s, is shown in Fig. 6a. The normalized fluctuation amplitude is found to be almost constant along the *z*-axis within the experimental uncertainties. However, the cross-correction between the total emissions indicates a significant value (> ~ 0.9), and the cross-correlation tends to decrease along the z-axis.

The fluctuation spectra calculated with the fast Fourier transform for the total plasma emissions show the coherent low-frequency fluctuations at *f* ≃ 3, 5, and 6 kHz, as shown in Fig. 6b. The axial cross-phase, coherence, and correlation length of the low-frequency fluctuations between axial positions with setting the reference position at *z* = 0.60 m are shown in Fig. 6c. The obtained coherence and correlation length reveal a significant value at the frequencies of the coherent mode fluctuations at *f* ≃ 3, 5, and 6 kHz to indicate the presence of the global coherent modes and local fluctuations. The correlation length, ${l}_{cor}$, which is defined as $C\left({z}_{i},{z}_{j}\right)={\epsilon }_{total,0}\mathrm{ exp}(-\left|{z}_{i}-{z}_{j}\right|/{l}_{cor})$, can distinguish the axial property of coherence more clearly in comparison with the scale of the axial length of the device. The comparable correlation length of the device means that the fluctuations should be global, whereas for the opposite case, the fluctuation should be local or turbulent.

Furthermore, the axial phase shows an interesting property that the phase delays are, surprisingly, almost the same at every observed frequency on both sides of the reference position at *z* = 0.60 m. This means that the fluctuations should propagate in both directions from the reference positions as the origin. In other words, the fluctuations should originate from a particular azimuthal position at *z* = 0.60 m. The characteristics are valid not only for the coherent modes but also for rather incoherent fluctuations. The detailed mechanisms of the interesting characteristics need to be examined.

## Summary

The 4-D measurement in time and space with the newly developed tomography system succeeded in disclosing unknown nature of plasma structure and dynamics in the linear magnetized plasma as follows: (1) detailed plasma evolution in the production and the relaxation phase, (2) presence of asymmetric part of emission profile at every axial position in the steady state with the persistence of the structure, and (3) the observation of 3-D origin for both coherent and incoherent fluctuations. The results demonstrate for the first time that the linear magnetized plasma should not be uniform along the axial direction and that asymmetric part of emission could be present in the perpendicular cross-section to the axial field despite its nominal symmetry. Finally, the 4-D tomography system of a new age allows to simultaneously examine the detailed dynamics of plasma structure in magnetized plasmas, and the initial results provide challenging subjects required to include inhomogeneity of structure and fluctuations into plasma physics of a new age.

## Methods

### Tomography measurement and algorithm

The tomography system can detect ArI and ArII emission signals as voltage with current–voltage converters (photodiode detectors) through optical filters. The wavelength band for ArII is used in this experiment, which is set at 450 nm ± 50 nm. The gain and frequency bandwidth of the detected emission signals are 10^8^ VA^−1^ and up to 50 kHz, respectively. The line-integrated raw data of emission signals are reconstructed into the local emission profile through the tomography algorithm named maximum likelihood–expectation maximization^35^, which is suitable for analyzing the plasma structure that frequently shows a discreate and abrupt change in space and time^27,36^. In this experiment, the raw signals of the two components are converted into local the emission profile on the values of the 121 (= 11 × 11) grids on a 160 mm × 160 mm square (responding to four sets of light-guide array), whereas the other raw signal is converted on the values of the 49 (= 7 × 7) grids on a 100 mm × 100 mm square (responding to six sets of light-guide array) in order to adjust the spatial resolution to the same size as the others for easy comparison.
